# Cost effectiveness of spread mitigation strategies for polyphagous shot hole borer *Euwallacea fornicatus* (Coleoptera: Curculionidae: Scolytinae)

**DOI:** 10.3389/finsc.2023.1279547

**Published:** 2023-12-18

**Authors:** David C. Cook, Peter S. Gardiner, Sonya Broughton

**Affiliations:** ^1^ Department of Primary Industries and Regional Development, Bunbury, WA, Australia; ^2^ School of Agriculture and Environment, University of Western Australia, Crawley, WA, Australia; ^3^ Harry Butler Institute, Murdoch University, Murdoch, WA, Australia; ^4^ Department of Primary Industries and Regional Development, South Perth, WA, Australia

**Keywords:** polyphagous shot hole borer, urban pest, pest management, cost effectiveness analysis, *Euwallacea fornicatus*

## Abstract

Polyphagous shot hole borer *Euwallacea fornicatus* Eichhoff was detected in Western Australia in September 2021, and an eradication campaign funded by the Commonwealth government is underway. As part of contingency planning, we examined the cost effectiveness of alternative control strategies that could be used to mitigate urban forest impacts and maintain the benefits of trees to the local communities if eradication was not feasible. At the time this work was undertaken, decision-makers were concerned about the potential need to replace all urban trees susceptible to attack. We considered this strategy alongside less destructive strategies and assessed their cost effectiveness in terms of material and labor costs and the loss of ecosystem services resulting from reduced tree foliage. Using a stochastic simulation model, we found that a strategy that involved pruning necrotic limbs and treating trees biennially with systemic insecticide was almost always more cost effective than removing infested trees and replanting to resistant varieties. We estimated this strategy would cost A$55-110 million over 50 years, while tree removal would cost $105-195 million. A third strategy using a mix of chemical suppression and tree removal was also considered in light of new information about the pest’s host preferences. With an estimated cost of $60-110 million, this strategy was only slightly more expensive than using chemical suppression alone and could actually lead to eradication if the host range is as narrow as recent survey data suggests.

## 1 Introduction

In September 2021, *Euwallacea fornicatus* Eichhoff was detected for the first time in Australia. It was discovered infesting a box elder maple tree (*Acer negundo* L.) in the Perth suburb of East Fremantle, Western Australia, and is now the subject of a national program to eradicate it from the Australian mainland. This analysis was originally undertaken as part of contingency planning in case eradication proved unsuccessful. In this hypothetical exercise, eradication was aborted, and the next best policy for biosecurity managers became a ‘slow the spread’ approach using different suppression policy options. Strategy A involved a relatively expensive one-off treatment in which infested trees were removed and replaced with non-host species (1), and strategy B involved a relatively cheap but on-going treatment in which infested trees were left standing, necrotic limbs removed and a systemic insecticide treatment administered every two years (2, 3). In this paper we describe how the cost effectiveness of each strategy was estimated and add in a third strategy, strategy C, that combines elements of the other two. We do this because of recent surveillance information that suggests the pest has a narrower preferred host range in Western Australia than it does elsewhere (4). Strategy C targets preferred trees for removal while using chemical suppressants on non-preferred hosts.

A member of the *E. fornicatus* species complex, *E. fornicatus* Eichhoff is commonly known as polyphagous shot hole borer (PSHB) and has gained notoriety for its effects on healthy and dead or dying urban forest trees in the last several decades. Globally, more than 680 species are susceptible to PSHB attack, 168 of which are thought suitable for reproduction (5). Damage occurs from beetles tunnelling into wood to create brood galleries and in the process introducing trees to infection from different fungi they farm as a food source for developing larvae inside the galleries, including *Fusarium euwallaceae* sp. nov. (6). This fungus causes localized tissue damage around attack sites (7, 8). The symbiotic fungus present in the Western Australian outbreak is currently referred to as *Fusarium* sp. [AF-18] (9), the binomial nomenclature having yet to be formalized.

When PSHB infestations are not managed, tree damage can be severe resulting in necrotic limbs and, in the most severe cases, tree death. In addition to lost aesthetic values (10), other public goods associated with trees are also affected, including noise, air and light pollution mitigation (11, 12), biodiversity (13) and carbon sequestration (14, 15). Private goods too are negatively affected by damage to neighborhood trees, including home and office real estate values (16) and temperature moderation (14). We assumed these costs would create a political imperative for state and local governments to employ suppression strategies to mitigate the impacts of PSHB if eradication was not technically feasible.

Delimiting surveys carried out as part of the national eradication program have thus far reported PSHB attacking almost 130 tree species in the Perth Metropolitan Area (4). These tree species are collectively referred to as “reproductive and non-reproductive hosts” (9), having been cross referenced against the international host literature. However, survey evidence suggests that reproduction may only be occurring in 10 “preferred hosts”, including box elder maple, mirror bush (*Coprosma repens*), poinciana (*Delonix regia*), coral tree (*Erythrina* x *sykesii*), Moreton Bay fig (*Ficus macrophylla*), Port Jackson fig (*Ficus rubiginosa*), white mulberry (*Morus alba*), black mulberry (*Morus nigra*), London plane tree (*Platanus* x *acerifolia*) and black locust (*Robinia pseudoacacia*) (9). It is possible that in the process of searching for these preferred hosts beetles attack alternatives situated nearby which are unsuitable for reproduction.

In the last 20 years, PSHB has become invasive in the U.S.A. (California, 2003), Israel (2009), South Africa (2017) and Palestine (2019) (17) where it has caused severe damage to urban forests (18–20). At the time of writing, information about *Fusarium* sp. [AF-18] is limited but it is presumed to have a similar effect on host trees as *F. euwallaceae*, only infecting tree tissue close to beetle attack points and rarely causing branch dieback (7, 8). It is likely that PSHB will spread slowly in Western Australia because only female*s* can fly and favor remaining in their natal branch rather than moving to new hosts (8). When females do fly, evidence from other members of the *Euwallacea fornicatus* species complex indicates short flight distances of <35 m are common, although occasionally longer flights of up to 400 m have been observed (21).

In this analysis, we used a cellular automata model to simulate the likely spread of PSHB through the Perth Metropolitan Area in the absence of an eradication program. A cellular automaton consists of a set of cell states within a lattice and a transition function for moving cells from one state to another. These models are particularly useful in the study of invasive species spread when data is limited (22). Our model was based on the land use change model described in Hewitt et al. (23) with the potential of cells to be infested depending on host presence/absence and Euclidian distance to other infested cells. We changed the rate of spread from a linear model to the logistic model of Cook and Broughton (24), and also raised the maximum percentage of hosts affected from 50-60% to 80-90% given the more precise tree data now available. This cellular automaton incorporates uncertainty about the rate of spread in the Perth region, with spread between neighboring grid cells prioritized while also incorporating satellite generation for intermittent spread to cells further afield.

Our prediction of the PSHB spread area over a 50-year planning horizon was used to assess the cost effectiveness of suppression strategies A, B and C in Perth’s urban forests. Cost effectiveness analysis compares the costs of policies designed to achieve the same outcome, with the lowest cost method generally being the one recommended. Although not as widely used as cost benefit analysis, which compares the net gains (or losses) produced by different policies, cost effectiveness analysis does not require an explicit quantification of benefits (25). Direct expenditures on materials and administration costs were included in our assessment, as well as indirect costs associated with environmental externalities. These externalities were estimated as ecosystem services using the i-Tree eco model (26), which captured carbon sequestration, storm water mitigation and air pollution removal. We omitted surveillance costs as they were assumed to be the same for each policy option, and therefore not critical to the outcome.

The paper adds to the growing PSHB literature by providing a quantitative assessment of suppression strategy costs. While other studies have estimated the costs of PSHB incursions in the extreme scenarios of minimal management (1) and eradication (24), to our knowledge no cost effectiveness studies have been completed for suppression or slow-the-spread strategies. The implications of uncertainty about PSHB host range in terms of suppression strategy costs may be of interest to policymakers in other regions tasked with making decisions about this important pest. It may also provoke discussion about response policies that do not necessarily involve large-scale tree removal in urban areas. All monetary values in the paper are provided in Australian dollars (AUD) unless otherwise stated.

## 2 Materials and methods

### 2.1 Study area and suppression strategies

**Figure 1:**
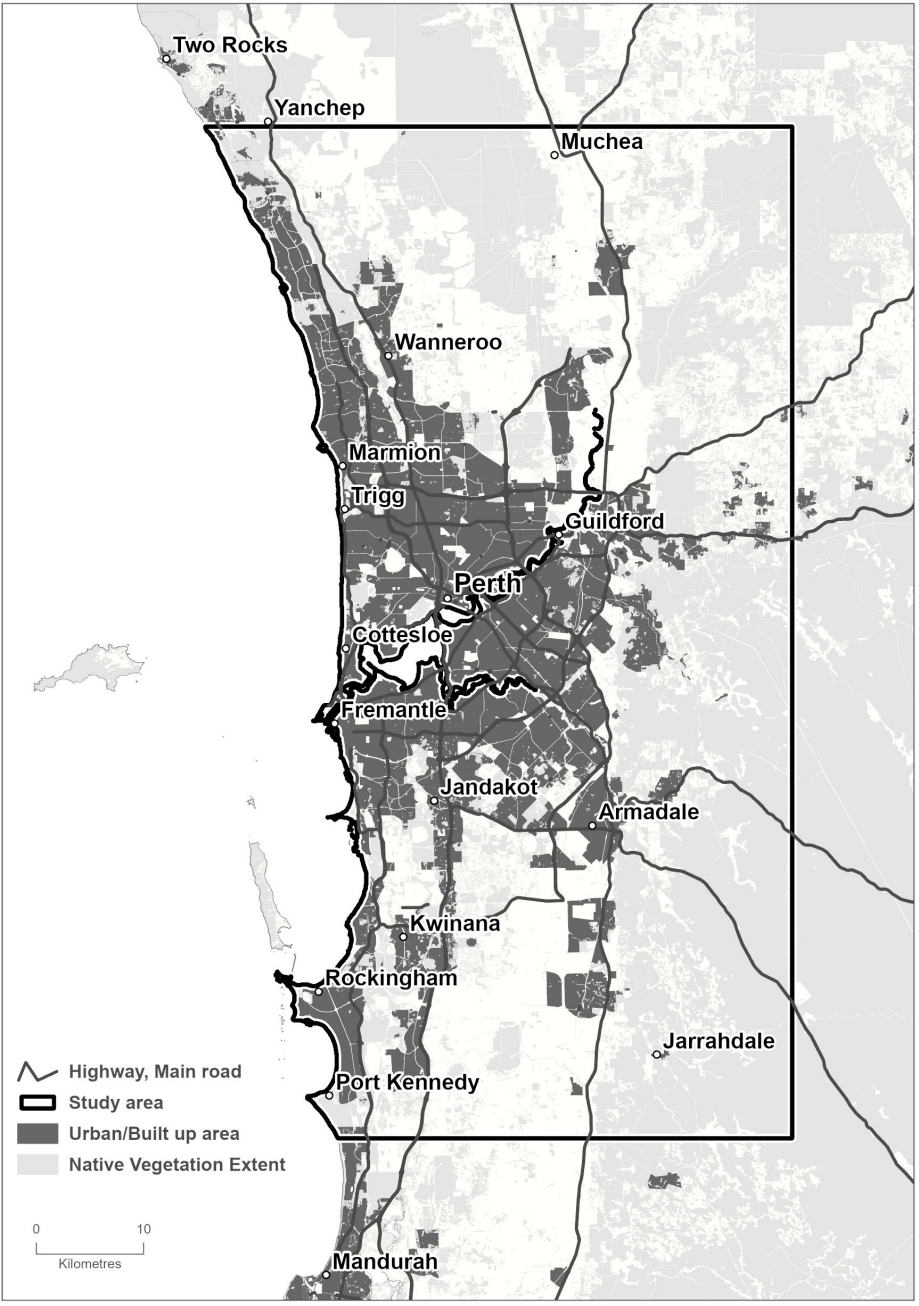
Perth Metropolitan Area. The study area is 412,535 ha, including the City of Perth, urban forest landscapes and native vegetation. It contains 142,288 host trees and more than 300 non-host species planted along roadsides, traffic islands, walkways, parks and gardens.

A section of the Perth Metropolitan Area in the South West Region of Western Australia was used as the study area (**Figure 1**). In total, this includes 412,535 ha, of which the Perth Central Business District occupies almost 2,000 ha. The climate in the South West region is Mediterranean, with cool wet winters and hot dry summers and is favorable to the establishment and spread of PSHB, which is considered to be native to Asia (17). Although the regional climate is changing, with average daily temperatures having increased 0.5°C and average rainfall having decreased 10-25% since 1975 (27), we assumed this will not affect the insect’s survival over the next 50 years. Ecologically, Perth is situated in the South West Botanical District, which is recognized as one of the world’s biodiversity hotspots (28). Native vegetation is dry sclerophyll, and ranges from heathlands, wetlands and open woodlands on the Swan Coastal Plain to tall eucalypt woodlands in the hills to the east (29). We assumed there is minimal risk to native species and that they would not require treatment in a suppression strategy, but it is difficult to be certain as evidence of PSHB damage elsewhere is mixed (24).

Over 300 native and non-native tree species are planted along roadsides, traffic islands, walkways, parks and gardens within the study area. Urban tree data from Department Of Primary Industries And Regional Development (4) provided total numbers, species and size information. The total number of host trees in the study area was 142,288, which included both reproductive (34%) and non-reproductive (66%) hosts reported at the time of writing (August 2023). This information was used as the basis for the wide host range scenario where all these trees would potentially be treated in suppression strategies A, B and C. These strategies are summarized in **Table 1**. We also considered a narrow host range scenario where only ten preferred host species were potentially treated, of which there are a total of 40,362 trees located in the study area. Delimiting survey information indicates these species are attacked most frequently by PSHB (9). In the hypothetical strategy C, which combined elements of both strategies A and B, we assumed that approximately 20% of preferred hosts were targeted for removal and replacement while all other reproductive and non-reproductive hosts were treated with systemic insecticides every two years after infestation. We assumed this sub-set was made up of box elder maple, coral tree and black locust trees, of which there are 8,214 in the study area. These four species were chosen as survey data places them at the center of attack clusters (4).

**Table 1:** Suppression strategies and scenarios.

	Wide host range scenario	Narrow host range scenario
Strategy A	All reproductive and non-reproductive hosts (142,288 trees) were susceptible. Treatment involved removing infested trees and replacing them with non-host tree species.	All preferred hosts (40,362 trees) were susceptible. Treatment involved removing infested trees and replacing them with non-host tree species.
Strategy B	All reproductive and non-reproductive hosts (142,288 trees) were susceptible. Treatment involved pruning necrotic limbs from infested trees and administering bell injections of systemic insecticide biennially.	All preferred hosts (40,362 trees) were susceptible. Treatment involved pruning necrotic limbs from infested trees and administering bell injections of systemic insecticide biennially.
Strategy C	All reproductive and non-reproductive hosts (142,288 trees) were susceptible. Treatment involved: (i) removing infested box elder maple, coral tree and black locust (8,214 trees) and replacing them with non-host tree species; (ii) pruning necrotic limbs from other infested trees and administering bell injections of systemic insecticide biennially.	All preferred hosts (40,362 trees) were susceptible. Treatment involved: (i) removing infested box elder maple, coral tree and black locust (8,214 trees) and replacing them with non-host tree species; (ii) pruning necrotic limbs from other infested trees and administering bell injections of systemic insecticide biennially.

### 2.2 Simulation model

A Susceptible-Infested-Resolved (S-I-R) model is used to describe total tree numbers affected and unaffected over time, where:


(1) $$S_{t}=S_{t-1}-\alpha I_{t-1}\left(\frac{S_{t-1}}{S_{0}}\right)$$

(2) $$I_{t}=I_{t-1}+\alpha I_{t-1}\left(\frac{S_{t-1}}{S_{0}}\right)-I_{t-1}$$

(3) $$R_{t}=R_{t-1}+I_{t-1}$$

(4) $$N=S_{t}+I_{t}+R_{t}$$

Here, *S_t_* refers to the number of susceptible trees within the study area in time step t ; $I_{t}$ is the number of infested trees in time step t ; $R_{t}$ is the number of resolved cases, or trees that are no longer susceptible to PSHB attack in time step t either because of replanting to resistant varieties or insecticide treatments; α is the average number of trees for which an infested tree will be the source of subsequent infestations; and N is the total number of host trees which is assumed constant.

Equation 1 states that the number of susceptible trees at time t  is equal to the number of susceptible trees in the previous time step minus newly infested trees. Equation 2 states that the number of trees acting as a source for infestation in period t is equal to the number of infested trees in the previous time step plus the number of newly infested trees minus the number of resolved cases. Resolved infestations are those that have either been removed or treated with insecticides. Equation 3 states that the number of trees previously infested with PSHB that are no longer a source of further infestations in time step t is equal to the number of resolved cases in the previous time step plus the number of resolved cases in the current time step. Finally, Equation 4 states the total number of host trees is equal to the sum of susceptible trees, infested trees and resolved cases.

Using the simulation output for I , costs involved in each response strategy were estimated. Because costs are incurred over time, they are subject to discounting. Discounting has an erosive effect on monetary values that increases with time, meaning that the same unit of cost incurred in the present is worth more than if incurred in the future. Assuming surveillance costs were the same in each suppression strategy and that no pre-emptive treatments were administered to susceptible trees, the discounted or present value of costs incurred under strategy i in time step t , $C_{it}$ , are:


(5) $$C_{it}=\frac{(I_{t}+{\hat{R}}_{t})\left[T_{i}+\omega_{i}W+\left(1-\beta_{it}\right)E\right]}{{\left(1+\upsilon \right)}^{t}}$$

Here, $\hat{R}$ is the number of resolved cases requiring recurrent treatment under strategy i in time step t ; $T_{i}$ is the capital cost of treating an infested tree under strategy *i; ω_i_* is the number of labor hours required to administer treatment to an infested tree under strategy i ; W is the average hourly wage rate of responders (assumed constant across strategies despite different skill requirements); *β_it_* is the rate of tree regrowth in time step t following response treatments prescribed in strategy i ; E is the ecosystem services produced by an average urban forest tree; and υ is the discount rate.

Equation 5 states that the cost of a response program is determined by the number of infested trees and resolved cases multiplied by the present value of material and labor costs involved in tree removal or pruning and insecticide treatment, plus the present value of ecosystem services lost.

Tree regrowth following replanting or pruning, $\beta_{it}$ Equation 6, was assumed to occur according to a Richards logistic growth function (30), such that:


(6) $$\beta_{it}=\frac{v^{\text{max}}}{1+\left(\frac{v^{\text{max}}}{v_{i}^{\text{min}}}-1\right)e^{-g\left(t-t^{*}\right)}}$$

where, $v^{\text{max}}$ is the maximum tree volume expressed in percentage terms; $v_{i}^{\text{min}}$ is the tree volume at replanting or following pruning expressed as a percentage of $v^{\text{max}}$ ; g is a constant rate of growth; and $t^{*}$ is the time step in which treatment is administered.

Parameter values for treatments appear in **Table 2** with explanations for each provided in the table notes. Using the Monte Carlo method parameters were specified as uniform distributions (with minimum and maximum values) or triangular distributions (with minimum, most likely and maximum values) when their specific values were not known. In all 10,000 iterations produced by the model, one value was randomly sampled from every distribution and those values were used to simulate spread and impact over the planning horizon. In this particular case, decision-makers chose a 50-year planning horizon.

**Table 2:** Parameters used in simulated polyphagous shot hole borer spread and management costs.

Parameter	Treatment	
	Removal and replanting	Pruning and bell injections
Area initially infested, $A^{\text{min}}$ (ha).*^a^*	1	1
Average number of trees for which an infested tree will be the source of subsequent infestation, α (#).*^b^*	Wide host range 1.3 Narrow host range 0.4	Wide host range 1.3 Narrow host range 0.4
Capital cost of treating an infested tree, T ($).*^c^*	Triangular(850,1500,4850)	37.5
Discount rate, υ (%).*^d^*	Uniform(3,7)	Uniform(3,7)
Ecosystem services produced by an average urban forest tree, E ($). ***^e^***	Uniform(2.3,5.2)	Uniform(2.3,5.2)
Hourly wage rate of responders, W ($).*^a^*	50	50
Labor hours required to administer treatment to an infested tree, ω (#).*^a^*	0.25	Triangular(1,2,3)
Maximum tree volume, $v^{\text{max}}$ (%).*^a^*	100	100
Minimum post-treatment tree volume, $v^{\text{min}}$ (%). *^a^*	Uniform(1,5)	Uniform(40,80)
Total number of host trees, N (#).*^f^*	Wide host range 142,288 Narrow host range 40,362	Wide host range 142,288 Narrow host range 40,362
Total number of susceptible trees at time t=0 , $S_{t_{0}}$ (#).*^a^*	Wide host range 142,287 Narrow host range 40,361	Wide host range 142,287 Narrow host range 40,361
Tree growth constant, g (%).*^g^*	Uniform(4,5)	Uniform(4,5)

^a^Plausible value.

^b^The average number of susceptible trees per hectare in the study area was 0.3 ha^-1^ in the wide host range scenario and 0.1 ha^-1^ in the narrow host range scenario (4). Given the relatively short flight distances observed in the *E. fornicatus* species complex (21), a square lattice was used with a 0.5 ha cell size and a Moore neighborhood structure (31), consisting of a source cell and the eight cells that surround it.

^c^Costs of tree removal and replacement provided in Treeswest (32). Costs of pruning and bell injections assumed biennial emamectin benzoate trunk injections (33) in conjunction with removal and disposal of necrotic limbs (24).

^d^Commonwealth Of Australia (34).

^e^USDA Forest Service (26).

^f^Department Of Primary Industries And Regional Development (4).

^g^Mcmahon and Parker (35).

### 2.3 Cellular automaton

A cellular automaton model based on the Simulation of Land-use Change Using R (SIMLANDER) package (23) was used to predict the spatial distribution of I over time on a map of urban forest areas in the Perth Metropolitan Area. A square landscape matrix with a cell size of 50 m^2^ was used with a Moore neighborhood structure (31), which consists of a central cell plus the eight cells that surround it. It is difficult to know how accurately this represents localized spread due to the absence of PSHB-specific data. Flight data for *E. perbrevis* Schedl, another member of the *E. fornicatus* species complex, suggests a Moore neighborhood structure will capture a majority of spread events as average flight distances are 30-35 m (21).

Host availability and proximity to infestations determined the probability a cell within the landscape grid would change from a susceptible to infested state in any given time step, and a randomized satellite site generator was also used to account for anthropogenic spread. This procedure involved several steps. Firstly, urban forestry maps were produced from the most recent data. Secondly, suitability maps were constructed based on the Euclidean distance from a source tree to each cell in the matrix. Thirdly, transition probability maps were formed based on changes in I and S in which cells containing susceptible trees closer to infested cells had higher probabilities of infestation in each successive time step. Finally, expansion maps were created showing the distribution of PSHB in each time step.

## 3 Results

### 3.1 Wide host range

**Figure 2:**
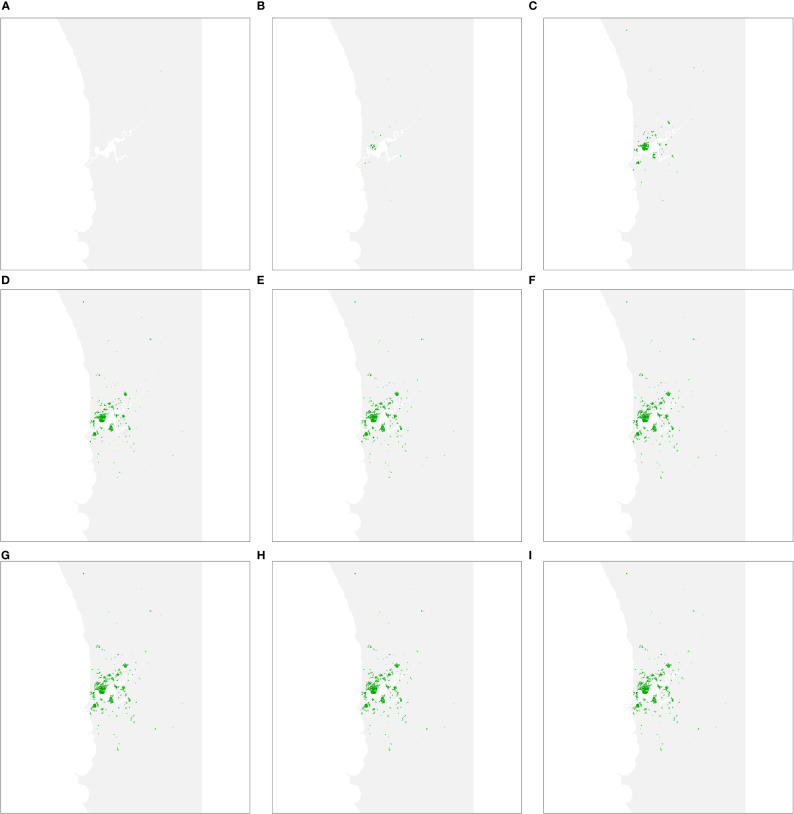
Predicted area of host trees affected over time if polyphagous shot hole borer has a wide host range. **(A–I)** show all resolved cases at time periods 5, 10, 15, 20, 25, 30, 40 and 50, respectively. Resolved cases are infested trees that have received treatment and are no longer susceptible to attack. Spread was most pronounced during the first 30 years of the simulation before slowing considerably as susceptible trees became scarcer.

As each management strategy was assumed to be equally successful in terms of mitigating impacts on urban forests, the simulated spread of PSHB in the absence of an eradication program was the same under strategies A, B and C. The first spread simulation we performed involved all hosts identified in Department Of Primary Industries And Regional Development (9), including both reproductive and non-reproductive hosts. **Figure 2** shows all resolved cases at different time intervals, where resolved cases are infested trees that received treatment and were no longer susceptible to attack. Spread occurred relatively quickly during the first 30 years of the simulation but slowed in the last 20 years as susceptible trees became scarcer.

**Figure 3:**
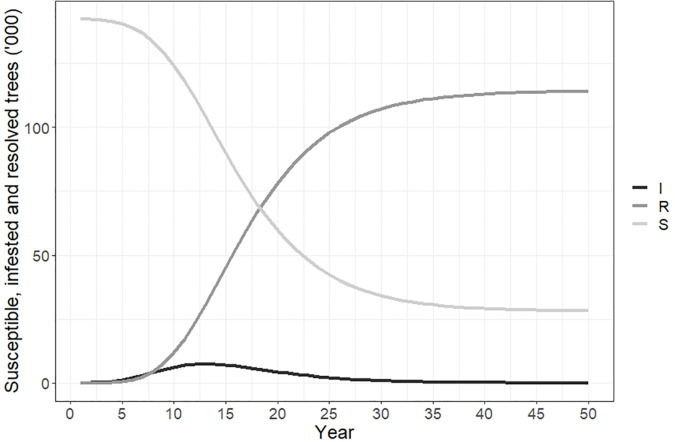
Susceptible, infested and resolved trees over time if polyphagous shot hole borer has a wide host range. Resolved cases are infested trees that have received treatment and are no longer susceptible to attack. The number of resolved trees increased rapidly between years 5-30 and reached a total of 120,490 by year 50. The number of trees susceptible to polyphagous shot hole borer attack fell to 21,800 over the same period, while the number of newly infested trees peaked in year 12 of the simulation at 9,250.

**Figure 3** plots the number of susceptible, infested and resolved cases over time. The total number of susceptible trees at the beginning of the simulation was 142,288, and by year 50 all but 21,800 of these had changed status. The number of resolved cases increased from 0 to 117,130 by year 30, and to 120,490 by year 50. This accounted for 84.7% of the total susceptible host area due to our modification to the Hewitt et al. (23) model to incorporate logistic spread and a maximum proportion of hosts infested of 80-90%. The number of newly infested trees peaked in year 12 at approximately 9,250.

**Figure 4:**
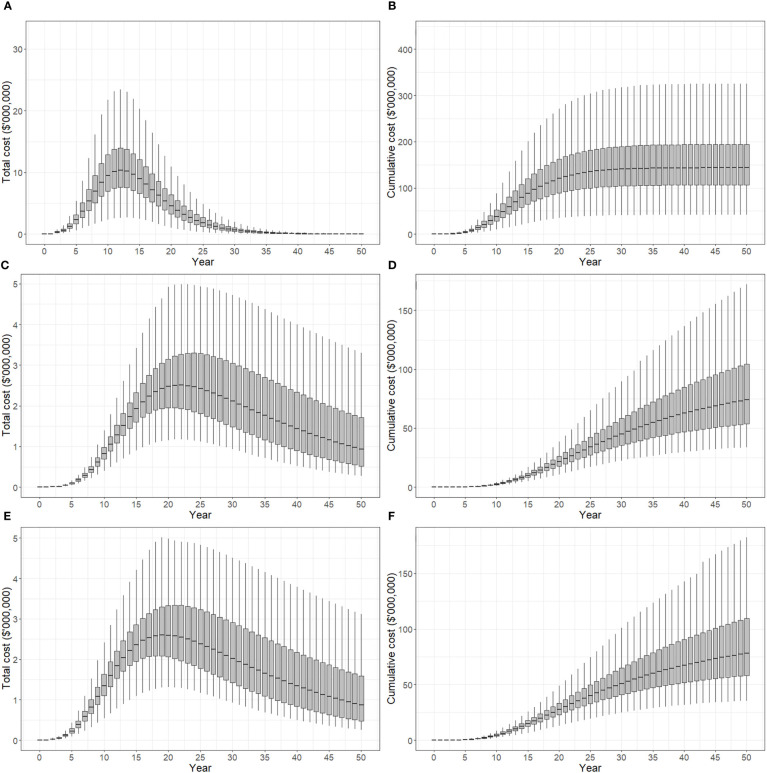
Total cost of control strategies if polyphagous shot hole borer has a wide host range. The plots show the 5^th^, 25^th^, median, 75^th^ and 95^th^ percentiles. **(A, B)** show the present value of total costs (annual) and cumulative costs incurred using strategy A (tree removal and replacement), respectively. **(C, D)** show the present value of total and cumulative costs incurred using strategy B (pruning and biennial insecticide injections), respectively. **(E, F)** show the present value of total and cumulative costs incurred using strategy C (mix of tree replacement and insecticide injections), respectively. Uncertainty in cost predictions increased with time, with cumulative costs over a 50-year planning horizon estimated to be A$105-195 million for strategy A, A$55-110 million for strategy B and A$60-110 million for strategy C.

Total costs and cumulative costs incurred under suppression strategies A, B and C are presented in **Figure 4**. Panels (A), (C) and (E) show total cost per year for each strategy, while panels (B), (D) and (F) show cumulative costs over time. All costs are presented in discounted, or present value terms. Discounting negatively impacts future monetary values, and the effect becomes stronger with time so that per unit costs are higher in the present than in the future. Strategy A produced a rapid acceleration in total cost between 0-12 years which peaked at approximately $10.5 million (median) before declining (panel A). Over the 50 years simulated in the model, 90% of iterations produced cumulative costs of between $105-195 million, while the median cost was approximately $145 million (panel B). Strategy B produced a more gradual change in total cost, which increased in successive time steps between years 5-22, and decreased from years 22-50 (panel C). After 50 years, 90% of model iterations produced cumulative costs between $55-105 million, and a median cost of approximately $75 million. Strategy C produced similar cost curves to strategy B, with total cost peaking slightly earlier in year 19 at $2.6 million (panel E), and cumulative costs after 50 years between $60-110 million (panel F). Median cumulative cost after 50 years was approximately $80 million.

### 3.2 Narrow host range

**Figure 5:**
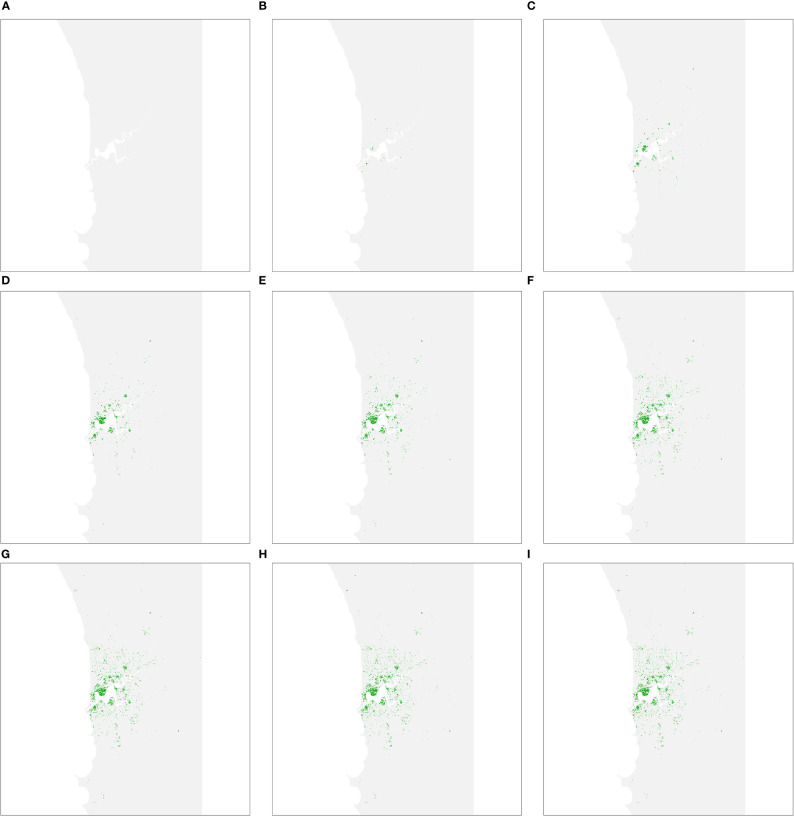
Predicted area of host trees affected over time if polyphagous shot hole borer has a narrow host range. **(A–I)** show all resolved cases at time periods 5, 10, 15, 20, 25, 30, 40 and 50, respectively. The distribution of resolved cases at respective time intervals was similar to the wide host range scenario as the ten most-preferred hosts are widely dispersed within the study area. The first 30 years of the simulation once again saw the most rapid expansion of cases before slowing.

The second spread simulation performed involved the narrow host range where only preferred hosts were considered. The number resolved cases at different time intervals are shown in **Figure 5**. The spread model parameters were held constant between both wide and narrow host range scenarios, so rapid spread was once again evident in the first 30 years of the simulation before slowing. However, as the number of host trees is considerably less (40,362, as opposed to 142,288 in the wide host range scenario), the infestation was more dissipated after 50 years than in the wide host range scenario.

**Figure 6:**
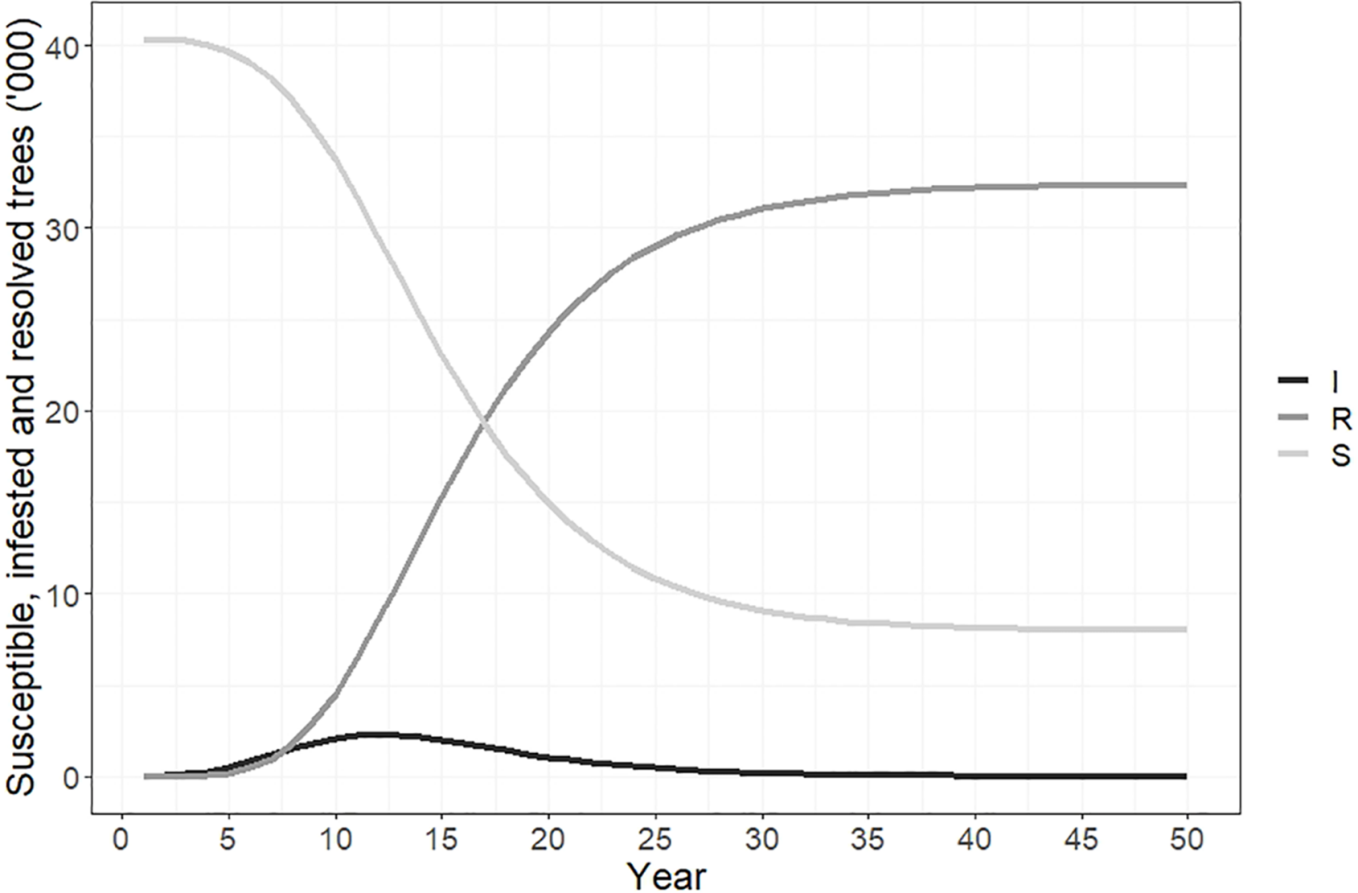
Susceptible, infested and resolved cases over time if polyphagous shot hole borer has a narrow host range. Resolved cases are infested trees that have received treatment and are no longer susceptible to attack. The number of resolved trees reached a total of 35,500 by year 50. The number of trees susceptible to polyphagous shot hole borer attack fell from 40,361 to approximately 5,400 over the same period, while the number of newly infested trees peaked in year 12 of the simulation at 2,490.

The number of susceptible, infested and resolved cases over time are shown in **Figure 6**. Susceptible cases fells from 40,361 to 5,400 over the 50-year simulation. Infested cases peaked in year 12 at approximately 2,500 trees before declining, and by year 50 the number of infested cases was <10. The number of resolved cases increased from 0 to 35,000 over 50 years.

**Figure 7:**
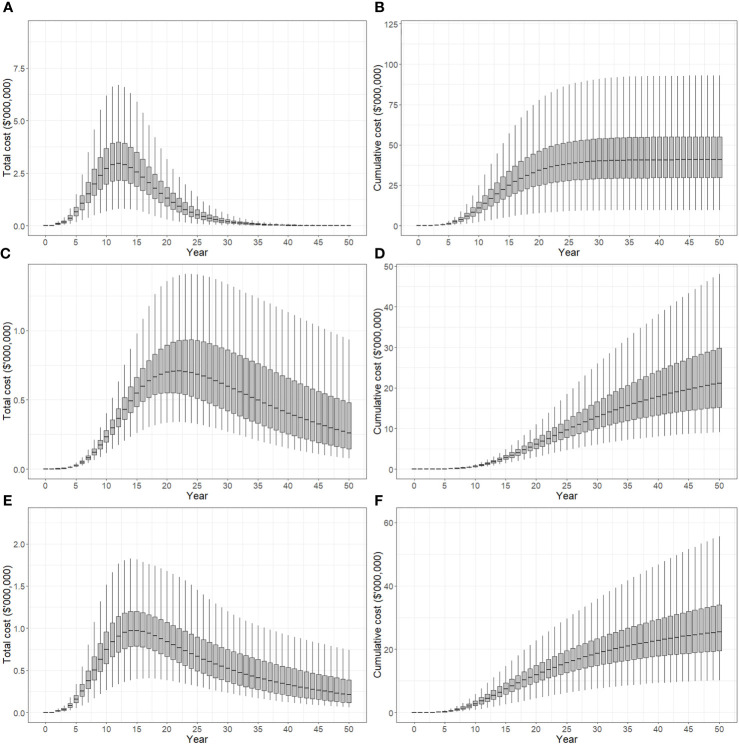
Total cost of control strategies if polyphagous shot hole borer has a narrow host range. The plots show the 5^th^, 25^th^, median, 75^th^ and 95^th^ percentiles. **(A, B)** show the present value of total costs (annual) and cumulative costs incurred using strategy A (tree removal and replacement), respectively. **(C, D)** show the present value of total and cumulative costs incurred using strategy B (pruning and biennial insecticide injections), respectively. **(E, F)** show the present value of total and cumulative costs incurred using strategy C (mix of tree replacement and insecticide injections), respectively. Cumulative costs over a 50-year planning horizon estimated to be A$30-55 million for strategy A, A$15-30 million for strategy B and A$20-35 million for strategy C.

Total and cumulative costs incurred under suppression strategies A, B and C appear in **Figure 7**. The total cost of strategy A increased between 0-12 years, reaching a maximum of $2.9 million, before declining (panel A). After 50 years, 90% of model iterations produced cumulative costs of between $30-55 million, while the median cost was approximately $41 million (panel B). The total cost of strategy B increased gradually between years 5-22 peaking at $0.7 million (median), and then decreased from years 22-50 (panel C). After 50 years, 90% of model iterations produced cumulative costs between $15-30 million, and a median cost of approximately $22 million. The total cost of strategy C peaked in year 14 at $0.9 million (panel E). Cumulative costs after 50 years were between $20-35 million with a median cost of approximately $26 million (panel F).

## 4 Discussion

In both the wide and narrow host range scenarios, strategy B was predominantly the most cost effective. Despite the recurrent pruning and chemical injection costs this strategy involved, cumulative costs over a 50-year planning horizon generally remained the lowest of the three strategies. Strategy A was the least cost-effective in both scenarios, with a cumulative cost almost double that of strategy B over 50 years. Although replacement with resistant tree varieties offered a permanent solution requiring no recurrent chemical treatment or pruning, the costs of tree removal were high. These costs also occurred early in the simulation period, corresponding to infestation cases surging within the first 20 years, and so were not eroded by the effects of discounting to the same extent as costs incurred later.

This result suggests that previous studies predicting the economic impacts of PSHB if unmanaged, which were the source of some consternation for Australian biosecurity policymakers when the insect was detected, may have overstated urban forest costs. Studies like De Wit et al. (20) and De Wit et al. (1), for example, drew much needed attention to an important pest. But assuming PSHB spreads rapidly through a broad range of hosts causing widespread tree deaths, and further assuming all affected urban forest trees would be removed inevitably results in extreme damage estimates. In our study, we presumed that PSHB would spread more slowly and could have a narrower host range in Western Australia than had been assumed in other models. If our assumptions are true, cheaper alternatives than removing infested trees may exist to manage PSHB in urban forests.

It is doubtful that, should it ever be needed, a slow-the-spread management strategy would only involve one specific treatment for all affected trees. Our results imply that the more emphasis placed on chemical treatments rather than tree removal, the lower the costs of an intervention will be. Strategy C, involving a mix of tree removal and chemical injection, was only slightly more expensive than strategy B as it targeted only preferred hosts. This approach could be further adapted to PSHB host preferences in Western Australia as further information comes to light, but the more trees that are targeted for removal, the further the strategy will be from the most cost effective option (strategy B).

Further exploration of the role surveillance activities could play in reducing overall treatment costs would be helpful if a slow-the-spread strategy is one day required. Surveillance activities were not specifically modelled in the current paper but were simply assumed to successfully identify newly infested trees within one year in all strategies. Surveillance efforts could slow PSHB spread more than we have assumed if the host range is narrow and monitoring activities are targeted at preferred hosts. Conversely, if the host range is larger and surveillance resources are spread more thinly across a wide range of tree species, spread could occur more quickly than we have assumed. These and other scenarios can be modeled in future by adapting the model to include detection uncertainty.

Our analysis did not consider logistical factors that might affect the viability of each strategy for decision-makers. Factors like labor constraints and chemical availability could limit the usefulness of a particular strategy in practice. Community perceptions of tree removal and chemical usage need to be carefully managed, particularly in a highly populated region like the Perth Metropolitan Area. Changes to urban landscapes alter residents’ aesthetic experiences, thereby influencing their perception of pest management strategies (36). So too can ethical, health and safety concerns around the use of toxins like pesticides (37–39). Demonstrating the cost effectiveness of different strategies, as the current study has done, can help to overcome these and other sources of social resistance (40).

Further on the issue of chemicals, the efficacy of the chemical suppression techniques proposed in Strategy B also warrants further investigation. The chemical we used in our costings (**Table 2**, note c), emamectin benzoate, is registered for use in Australia but has not been trialed in the control of PSHB (33). It has proved an effective PSHB suppressant in California (3, 41, 42), and has also been shown to be effective in the control of other boring beetles (43–45). However, we acknowledge that trunk injections of this or any other chemical have yet to be tested in Western Australia to control PSHB, and the effectiveness of this strategy in different hosts is unknown.

We note that while cellular automaton models like the one used in this analysis have the advantage of simplicity, they do have several shortcomings. As they delineate landscapes to spatial units, they are not well suited to dealing with diverse landscapes or environments where movement probability varies according to exogenous factors like wind or water dispersion (46). The rules-based spread mechanism between cells in the lattice also makes it difficult to incorporate complex invasive species behaviors. Alternatives, such as species dispersal models (47–49), are better suited to larger spatial or temporal scales, or more nuanced case studies. These models incorporate the processes regulating species survival, reproduction and movement in response to local environment conditions (49), but their reliability when used for prediction has been widely debated (50). Simpler mechanistic spread models that incorporate a dispersal kernel, such as reaction–diffusion models (51–53), can also be useful in simulating invasive species impacts over large areas, but lack geographical inputs that can affect species movement over time, particularly in slow-spreading cases like PSHB.

Having map-based outputs, the model used in this study can facilitate stakeholder communication. Decision-makers are usually time-poor and seldom have experience in developing simulation models, so it is often difficult for them to invest the time necessary to understand a particular decision-support tool enough to trust in its output. Our model is intended to be used interactively. The simple spread projection (**Figures 2**, **5**), for example, can be viewed in real time, enabling decision-makers to quickly grasp the spatial and temporal dimensions of spread, while the economic indicators (**Figures 4**, **6**) communicate financial implications and uncertainty. Interaction with these model outputs also creates a feedback loop with decision-makers, helping them, for example, to specify the planning horizon they wish applied to the policy choice. Although not critical in this case, where strategy B is superior to the alternatives over a long planning horizon like 50 years, in other settings the most cost-effective option may depend on the timeframe decision-makers are interested in.

Although we have included lost carbon sequestration benefits in our estimation of costs under different PSHB suppression strategies, other environmental costs should also be taken into consideration. As a global biodiversity hotspot, the South West Botanical District contains flora and species interdependencies that are unique, and in many cases, poorly understood (28). Evidence seems to indicate minimal impacts from PSHB on native species, at least in urban forests, so we have not considered costs of suppression measures extending to native forest areas or environmental costs associated with machinery and insecticide usage. The impact the borer and *Fusarium* sp. [AF-18] might have in these areas remains unclear (5), but if native species were to be affected we would need to revise the tree numbers used in the assessment to give a better indication of the cost of each strategy. Similarly, we have assumed suppression strategies will target tree species regardless of the disservices they might impose on communities. Preventing damage to some trees could increase potential storm damage, local flooding and fire risk (54). Moreover, as they are all non-native trees that potentially provide food and shelter for other introduced species, there may be additional costs associated with their protection that we have not accounted for.

The situation with PSHB in Western Australia is evolving, and information from field operatives will be critical in improving our model given the unique PSHB-*Fusarium* sp. symbiosis discovered. Many tree species have been observed with beetles and/or *Fusarium* sp. [AF-18] present (4), but it is less clear which trees are suited to life cycle completion. Should reproduction only prove possible in box elder maple, coral tree and black locust, as in our example strategy C, with other trees located nearby only receiving superficial damage, then the replacement of these species will eventually see the PSHB population collapse. If this is the case, a strategy resembling strategy C would produce the same result as the current eradication program at approximately half the cost (**Figure 7F**), and may even be cheaper if insecticide treatments in other trees are not necessary. At the time of writing, the eradication program is set to take place over 3 years with an estimated cumulative cost of A$45 million (24). Of course, our costings relate to suppression strategies rather than eradication, and thus omit additional costs associated with eradication programs, including the extensive surveillance effort that has generated the data used in the analysis.

## Data availability statement

The original contributions presented in the study are included in the article/supplementary files, further inquiries can be directed to the corresponding author/s.

## Author contributions

DC: Conceptualization, Formal analysis, Investigation, Methodology, Visualization, Writing – original draft. PG: Conceptualization, Data curation, Visualization, Writing – original draft. SB: Conceptualization, Writing – original draft.
